# Altered Mental Status in the Emergency Department – When to Consider Anti-LGI-1 Encephalitis: Case Report

**DOI:** 10.5811/cpcem.2021.4.51535

**Published:** 2021-07-27

**Authors:** Stephanie S. Miljkovic, B. Witkind Koenig

**Affiliations:** Creighton University School of Medicine, Department of Emergency Medicine, Phoenix, Arizona

**Keywords:** Autoimmune encephalitis, altered mental status, LGI-1 antibody, limbic encephalitis

## Abstract

**Introduction:**

Anti-leucine-rich glioma inactivated-1 (LGI-1) is one of few antibodies implicated in limbic encephalitis, a syndrome that can result in permanent neurological symptoms if left untreated.

**Case Report:**

We present a patient with dystonic seizures, progressive cognitive decline, psychiatric symptoms and short-term memory loss, and mild hyponatremia diagnosed with anti-LGI-1 antibody limbic encephalitis.

**Conclusion:**

There are few reports in the emergency medicine community describing anti-LGI-1 antibody limbic encephalitis. Delay in diagnosis can risk irreversible limbic damage. Therefore, it is important for the emergency physician to be aware of anti-LGI-1 antibody limbic encephalitis when presented with adult-onset seizures and altered mental status of unknown etiology.

## INTRODUCTION

Limbic encephalitis (LE), a subtype of autoimmune encephalitis, is characterized by an antibody-mediated inflammation of the limbic region. The limbic system is comprised of the hippocampus, hypothalamus, cingulate gyrus, amygdala, and limbic cortex. Together, these structures play a crucial role in one’s emotions, learning, memory, and motivation. In essence, the limbic system significantly contributes to a person’s temperament. Limbic encephalitis is classically recognized as a paraneoplastic disease, associated most often with small-cell carcinoma of the lung, various testicular tumors, thymoma, breast cancer, and Hodgkin lymphoma. Nevertheless, it is now understood that LE can also manifest secondary to an autoimmune or post-infectious process. Patients typically present with dystonic seizures, altered mental status, and other neurologic deficits related to limbic function. While LE is potentially reversible with early immunotherapy, failure to recognize the condition can lead to irreversible disease progression and poor neurological outcomes. We discuss a patient who presented to the emergency department (ED) with progressive cognitive decline, seizures, memory loss, and emotional instability who was ultimately diagnosed with anti-LGI-1 antibody LE.

## CASE REPORT

A 70-year-old, Spanish-speaking female with a two-month history of newly diagnosed Parkinson’s disease, anxiety, and complex partial seizures, who was recently started on valproic acid, presented to our ED in the company of her daughter for evaluation of increasing seizure episodes and confusion in the prior 24 hours. The patient denied any infectious symptoms, headache, or back pain. Her daughter noted that the patient had been high functioning, serving as the primary caregiver for her own mother until two months earlier, when she abruptly developed progressive short-term memory deficits, decreased appetite, insomnia, severe paranoia, hallucinations, and episodes of stiffening and jerking movements of the face and arms that had worsened, despite initiation of valproic acid. Her past medical history was significant for polyps on her last colonoscopy. Her surgical, social, and family histories were negative for neurological diseases besides the aforementioned recent diagnoses.

On examination, her vital signs were unremarkable. Despite her confusion, she was alert and oriented with clear speech in Spanish when questioned, but she did not participate in history taking. Her pupils were normal. The patient’s right face tensed for approximately 10 seconds followed by mild confusion, but she could follow commands appropriately afterward without an obvious postictal period. No other focal neurological deficits were appreciated on neurological exam.

Her basic metabolic panel was significant for mild hyponatremia (133 millimoles per liter [mmol/L] (reference range 136–145 mmol/L). Her complete blood count was within normal limits. Despite abnormal urinalysis, her urine cultures were negative for growth. Her cerebrospinal fluid (CSF) protein (280.4 milligrams per deciliter [mg/dL] (reference range 15–60 mg/dL) and CSF red blood cell count (260,000/ microliter, normal = 0) were moderately elevated but more likely attributed to a traumatic lumbar puncture. Her CSF Gram stain was negative.

Non-contrast head computed tomography (CT) and magnetic resonance imaging (MRI) showed multiple abnormalities. Her CT was significant for scattered calcifications in the right caudate, right insular region, and right frontal lobe, which were reported as non-specific findings, possibly representing calcified emboli. Her MRI, which was obtained inpatient, demonstrated right amygdala and anterior temporal lobe edema and hyperintense thickening on the fluid-attenuated inversion recovery (FLAIR) sequence (Image).

Once autoimmune encephalitis was suspected, serum specimens were sent and resulted positive in less than one month. The inpatient encephalopathy, autoimmune evaluation, and spinal fluid 2 assay confirmed the anti-LGI-1 antibody in her CSF. This finding, along with hyponatremia, faciobrachial dystonic seizures, and hyperintense limbic thickening on MRI (Image) were all consistent with LGI-1 antibody encephalitis. The patient began immunotherapy treatment with rituximab and a prednisone taper. On follow-up, she has reportedly demonstrated a robust response with near-resolution of symptoms. The presence of occult malignancy is under investigation at this time, with a chest radiograph without evidence of malignancy, and a pending colonoscopy.

**CPC-EM Capsule**

What do we already know about this clinical entity?

*Autoimmune limbic encephalitis is a rare cause of adult-onset seizures and altered mental status with associated memory loss, cognitive decline and psychiatric manifestations*.

What makes this presentation of disease reportable?

*Our patient had anti-LGI-1 antibody encephalitis characterized by a constellation of faciobrachial dystonic seizures, memory loss, emotional lability, and psychiatric disturbances*.

What is the major learning point?

*In the setting of acute adult onset seizures, cognitive decline, and psychiatric manifestations, emergency physicians should have a high degree of suspicion for an autoimmune encephalitis*.

How might this improve emergency medicine practice?

*Early recognition and appropriate treatment of autoimmune encephalitis in the emergency department has the opportunity to reverse existing limbic damage with complete resolution of symptoms*.

## DISCUSSION

Autoimmune encephalitis is an uncommon cause of acute altered mental status and new-onset seizures in the ED. While there is an exhaustive list of antibody targets implicated in autoimmune encephalitis as a whole (ie, N-methyl-D-aspartate receptor, α-amino-3-hydroxy-5-methyl-4-isoxazolepropionic acid receptor), only a few proteins have been identified that are specifically targeted in the limbic system, namely leucine-rich glioma inactivated-1 (LGI-1) and contactin-associated protein-like 2 (CASPR2) membrane protein.^1^ These two proteins are functional components of the voltage-gated potassium channels (VGKC) in limbic neurons.^2^ While the pathophysiology of LE is not completely understood, it is thought that the antibodies targeting LGI-1 and CASPR2 disrupt the function of VGKCs, leading to hyperexcitability of neurons found in the limbic system.^1^ While LE as a cause of autoimmune encephalitis is uncommon, anti-LGI-1 antibody subtype of LE is rarer still, with an estimated annual incidence of .83 cases/million.^3^

Antibody production in LE was once thought to arise solely in the context of paraneoplastic disease; however, individual case reports in the last decade demonstrate anti-LGI-1 antibodies in the CSF without an underlying malignancy. Anti-LGI-1 antibody LE is exceedingly rare. Thus, LE can arise from both paraneoplastic and independent autoimmune or post-infectious processes, the latter now clinically presenting more frequently in current literature.^1^ Given our patient’s recent colonoscopy demonstrating polyps, it would not be unreasonable to consider further investigation for an underlying neoplastic process to explain a potential paraneoplastic etiology of her LE.

Limbic encephalitis is commonly preceded by episodic seizures known as faciobrachial dystonic seizures (FBDS).^4^ Emergency physicians may recognize these uncommon seizures as brief (ie, seconds), unilateral facial grimacing and arm posturing, as were seen in our patient on physical exam.^5^ The presentation of FBDS are characteristically adult-onset, refractory to anti-epileptic drugs, and immunosuppressive-therapy responsive.^6^ Although highly associated with the LGI-1 antibody, it is unclear whether presence of FBDS is rather a symptom than a cause of the development of LE.^5^ Nevertheless, emergency physicians may need to consider anti-LGI-1 antibody LE should these rare but classic seizures be observed.

Additionally, limbic involvement in autoimmune encephalitis should present with one or more of the following psychiatric features: emotional instability; psychosis; memory loss; cognitive decline; or sleep-related disorders. Our patient struggled with all of these symptoms at various times in her illness. The delay in diagnosis of LE can be reasonably attributed to the overlap of psychiatric findings found in more prevalent geriatric conditions, including dementia, substance use, delirium, and other primary mood disorders. Lastly, imaging and CSF analysis play a supportive role to further delineate a diagnosis of anti-LGI-1 antibody LE.^6^ Patients suspected of the disease will typically present with either an additional finding of a neoplastic mass, a confirmed anti-LGI-1 antibody on CSF encephalopathy-autoimmune evaluation, and/or an increased FLAIR hyperintense signaling in the medial temporal lobes on MRI (unilaterally or bilaterally).^7^

In a case such as the one we report here, it is unlikely that an emergency physician would include MRI in the initial workup; rather, a head CT would more likely be performed. However, CT would probably only demonstrate calcifications that are largely non-specific and more representative of chronic changes common to our patient’s age group, raising little suspicion for further investigation. Unfortunately, if left untreated, most patients progress to permanent hippocampal atrophy with irreversible cognitive dysfunction.^8^ Additionally, hyponatremia has been found to be a characteristic finding of LE, although non-specific and associated with many seizure disorders.^9^

One month prior to her visit to the ED, our patient was initially misdiagnosed with parkinsonism, and generalized anxiety. Nevertheless, her facial and upper extremity seizures were refractory to valproic acid, and her hallucinations and cognitive decline worsened despite the increased dosing of haloperidol and quetiapine. The delay in diagnosis is typical of patients with autoimmune encephalitis. Although autoimmune encephalitis of any kind is ultimately not an ED diagnosis, it is crucial for the emergency physician to recognize this constellation of FDBS, hyponatremia, and worsening cognitive decline as being highly suspicious of anti-LGI-1 antibody LE. The physician should obtain CSF studies and MRI to evaluate the limbic region if the diagnosis is possible, as early diagnosis and treatment of LE can prevent or reverse existing hippocampal damage and cognitive decline.^9^

The first-line treatment of LE includes corticosteroids, intravenous immunoglobulins, and plasmapheresis. These treatment options have shown clinical improvement in up to 80% of patients.^10^ The second-line treatments include the addition of immunosuppressive therapy, such as mycophenolate mofetil or rituximab, although the benefits of these options are still not clear.^11^ If the diagnosis is being considered, neurology consultation will help guide treatment choice.

## CONCLUSION

The adult patient with acutely altered mental status and newly diagnosed seizures in the absence of clear etiology presenting to the ED should be considered for workup of autoimmune encephalitis. Emergency physicians encountering the additional findings of faciobrachial dystonic seizures, hyponatremia, and MRI abnormalities in the limbic region should have high clinical suspicion for anti-LGI-1 antibody limbic encephalitis. Confirmatory tests include anti-LGI-1 antibodies in the cerebrospinal fluid. Early diagnosis and treatment with first-line agents have a high likelihood of reversal of cognitive decline and permanent limbic damage.

## Figures and Tables

**Image:**
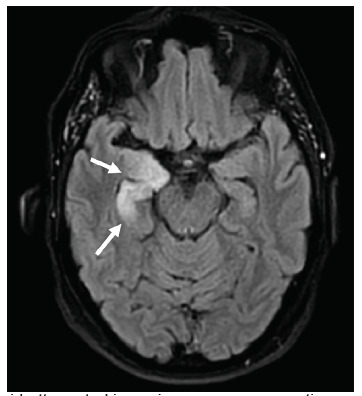
Fluid-attenuated inversion recovery magnetic resonance imaging demonstrating asymmetric swelling and edema within the right hippocampus (white arrows) suggestive of limbic encephalitis.

## References

[1] Wang M, Cao X, Liu Q (2017). Clinical features of limbic encephalitis with LGI1 antibody. Neuropsychiatr Dis Treat.

[2] Vincent A, Buckley C, Schott JM (2004). Potassium channel antibody-associated encephalopathy: a potentially immunotherapy-responsive form of limbic encephalitis. Brain.

[3] van Sonderen A, Thijs RD, Coenders EC (2016). Anti-LGI1 encephalitis: clinical syndrome and long-term follow-up. Neurology.

[4] Andrade DM, Tai P, Dalmau J (2011). Tonic seizures: a diagnostic clue of anti-LGI1 encephalitis?. Neurology.

[5] Irani SR, Michell AW, Lang B (2011). Faciobrachial dystonic seizures precede Lgi1 antibody limbic encephalitis. Ann Neurol.

[6] Thompson J, Bi M, Murchison AG (2018). The importance of early immunotherapy in patients with faciobrachial dystonic seizures. Brain.

[7] Navarro V, Kas A, Apartis E (2016). Motor cortex and hippocampus are the two main cortical targets in LGI1-antibody encephalitis. Brain.

[8] Heine J, Prüss H, Kopp UA (2018). Beyond the limbic system: disruption and functional compensation of large-scale brain networks in patients with anti-LGI1 encephalitis. J Neurol Neurosurg Psychiatry.

[9] Broadley J, Seneviratne U, Beech P (2018). Prognosis in autoimmune encephalitis: Database. Data Brief.

[10] Wong SH, Saunders MD, Larner AJ (2010). An effective immunotherapy regimen for VGKC antibody-positive limbic encephalitis. J Neurol Neurosurg Psychiatry.

[11] Titulaer MJ, McCracken L, Gabilondo I (2013). Treatment and prognostic factors for long-term outcome in patients with anti-NMDA receptor encephalitis: an observational cohort study. Lancet Neurol.

